# Reduced brain activity during a working memory task in middle-aged apolipoprotein E ε4 carriers with overweight/obesity

**DOI:** 10.3389/fnhum.2022.1001229

**Published:** 2022-11-25

**Authors:** Jermon A. Drake, John M. Jakicic, Renee J. Rogers, Sarah L. Aghjayan, Chelsea M. Stillman, Shannon D. Donofry, Kathryn A. Roecklein, Wei Lang, Kirk I. Erickson

**Affiliations:** ^1^Department of Psychology, University of Pittsburgh, Pittsburgh, PA, United States; ^2^Center for Neural Basis of Cognition, Carnegie Mellon University, Pittsburgh, PA, United States; ^3^Division of Physical Activity and Weight Management, Department of Internal Medicine, University of Kansas Medical Center, Kansas City, KS, United States; ^4^Wondr Health, Inc., Dallas, TX, United States; ^5^Allegheny Health Network, Psychiatry and Behavioral Health Institute, Pittsburgh, PA, United States; ^6^Center on Aging and Mobility, University Hospital Zurich, University of Zurich, Zurich, Switzerland; ^7^PROFITH “PROmoting FITness and Health Through Physical Activity” Research Group, Department of Physical and Sports Education, Faculty of Sport Sciences, Sport and Health University Research Institute (iMUDS), University of Granada, Granada, Spain; ^8^AdventHealth Research Institute, Neuroscience Institute, Orlando, FL, United States

**Keywords:** APOE, fMRI, cerebral blood flow (CBF), obesity, working memory

## Abstract

**Objective:**

The apolipoprotein E ε4 (APOE ε4) allele and midlife obesity are independent risk factors for Alzheimer’s disease (AD). Both of these risk factors are also associated with differences in brain activation, as measured by blood oxygenation level-dependent (BOLD) responses, in the absence of detectable cognitive deficits. Although the presence of these risk factors may influence brain activity during working memory tasks, no study to date has examined whether the presence of the ε4 allele explains variation in working memory brain activity while matching for levels of overweight/obesity. The primary aim of this study was to determine whether the presence of the ε4 allele is associated with differences in task-functional magnetic resonance imaging (fMRI) brain activation in adults with overweight/obesity. We predicted that ε4 carriers would have greater brain activation in regions that support working memory.

**Methods:**

This ancillary study included 48 (*n* = 24 APOE ε4 carriers; *n* = 24 APOE ε4 non-carriers), sedentary middle-aged adults (Mean age = 44.63 ± 8.36 years) with overweight/obesity (Mean BMI = 32.43 ± 4.12 kg/m^2^) who were matched on demographic characteristics. Participants were a subsample enrolled in 12-month randomized clinical trial examining the impact of energy-restricted diet and exercise on cardiovascular health outcomes. Participants completed a n-back working memory task with fMRI, which were completed within one month of the start of the intervention. Participants also underwent pseudo-continuous arterial spin labeling scans, a MRI measure of cerebral blood flow (CBF).

**Results:**

Compared to non-ε4 carriers with overweight/obesity, ε4 carriers with overweight/obesity had lower fMRI brain activity in the middle frontal gyrus, pre and post central gyrus, supramarginal gyrus, superior temporal gyrus, lateral occipital cortex, and angular gyrus (*z* range = 2.52–3.56) during the n-back working memory task. Differences persisted even when controlling for CBF in these brain regions.

**Conclusion:**

These results indicate that presence of the APOE ε4 allele in middle-aged adults with overweight/obesity is related to altered brain activity during a working memory paradigm, which may confer risk for accelerated neurocognitive decline in late adulthood. Future research is needed to clarify the clinical implications of these findings in the context of risk for AD.

## Introduction

The apolipoprotein E (APOE) ε4 allele is associated with risk for accelerated neurocognitive decline (Espeseth et al., 2008; Whitehair et al., 2010; Xu et al., 2013; Rieckmann et al., 2016) and alterations in cerebrovascular function (Thambisetty et al., 2010; Wierenga et al., 2013, Brandon et al., 2018; Hays et al., 2019). In addition, the ε4 allele is also considered the primary genetic risk factor for late-onset Alzheimer’s disease (AD) (Sando et al., 2008; Liu et al., 2014; Di Battista et al., 2016). The frequency of the ε4 allele in the population ranges from 9 to 23% (Bertram et al., 2007; Jia et al., 2020), and relative to ε3 homozygotes, individuals that possess one ε4 allele are one to four times more likely to develop AD, and individuals with two ε4 alleles are on average 15 times more likely to develop AD (Farrer et al., 1997). AD-related pathology, such as amyloid-beta (Aβ), begins to form 10 to 20 years or several decades before the onset of behavioral and neurocognitive deficits, and is more prevalent in ε4 carriers in midlife (Bateman et al., 2012; Sperling et al., 2014; Stocker et al., 2020). For example, in a post-mortem sample of individuals who died from non-AD-related causes, Kok et al. (2009) found that 40.7% of ε4 carriers aged 50–59 had at least one Aβ plaque, compared to only 8.2% of non-carriers in the same age range. Such findings suggest that it may be possible to detect changes in neurological health in midlife, well before AD symptom manifestation, especially among those at a greater genetic risk for developing AD.

One method for detecting preclinical brain changes is through the use of functional magnetic resonance imaging (fMRI) (Wagner, 2000). Cross-sectional fMRI studies suggest that cognitively normal carriers of the ε4 allele, ranging from younger to older adults, have altered functional brain activation during several cognitive tasks such as working memory, episodic memory, semantic, naming, and self-appraisal tasks (for review see Trachtenberg et al., 2012). More specifically, studies conducted in middle-aged or older adults have shown that cognitively normal ε4 carriers have elevated neural activity during cognitive tasks relative to non-carriers in frontal, parietal, and temporal regions, even in the absence of reduced cognitive performance (Bookheimer et al., 2000; Wishart et al., 2006; Chen et al., 2013; Scheller et al., 2017). This finding has been interpreted as evidence that ε4 carriers must compensate for early-stages of developing neuropathology by recruiting additional neuronal resources to perform similarly to non-carriers on cognitive tasks (Bondi et al., 2005; for review see Han et al., 2009). However, few studies have taken into account individual differences in other factors that could be influencing blood oxygenation level-dependent (BOLD) responses, which captures localized changes in deoxyhemoglobin concentrations, such as variation in resting state cerebral blood flow (CBF) and overweight/obesity. Here, we take advantage of multi-modal neuroimaging methodologies to address this gap in our mechanistic understanding of the relationship between ε4 and task-fMRI brain function.

Differences in, or changes to, task-fMRI BOLD signal are attributable to alterations in neuronal activity through cerebrovascular coupling (Gore, 2003). Since the fMRI response is an indirect measure of neuronal activity that is dependent upon CBF (Havlicek et al., 2017) and the ε4 allele affects cerebrovascular function (Thambisetty et al., 2010; Wierenga et al., 2013, Brandon et al., 2018; Hays et al., 2019), it is plausible that genetic variation in CBF is confounding interpretations of fMRI signal differences between ε4 carriers and non-carriers (Fleisher et al., 2009; Han et al., 2009; Trachtenberg et al., 2012). As such, controlling for differences in resting CBF could help clarify whether differences in task-fMRI patterns between ε4 carriers and non-carriers are reflective of differences in task-fMRI neural activity or CBF.

Obesity might be another important factor to consider when evaluating brain activation differences between ε4 carriers and non-carriers. Obesity in midlife has been associated with a higher prevalence of cognitive impairment and dementia in late adulthood, including AD (Gunstad et al., 2010; Profenno et al., 2010; Luchsinger et al., 2012), with weight-related decrements in cognitive performance emerging decades before the development of overt pathology. This is alarming given that, according to data from the 2015 to 2016 National Health and Nutrition Examination Survey, nearly 40% of adults in the United States have obesity (Hales et al., 2017), with more than 1.9 billion adults meeting clinical criteria for overweight or obesity worldwide (World Health Organization [WHO], 2018). Yet research exploring how measures of overweight/obesity status might be confounding interpretations of fMRI results in ε4 carriers has not been considered in prior studies.

Although both the ε4 allele and obesity confer risk for cognitive decline and dementia, the relationship between these factors and how they interact to affect brain function is complex and not well understood. For instance, studies suggest that ε4 carriers tend to have a lower body mass index (BMI) than non-ε4 carriers. Yet, ε4 carriers appear to be more impacted by excess body fat (Jones and Rebeck, 2018). For example, compared to non-carriers with excess body fat, ε4 carriers with excess body fat have been found to have elevated levels of glucose and insulin (Elosua et al., 2003). Further, the relationship between proxy measures of body fat (i.e., BMI and waist circumference) and brain function is in the opposite direction of that observed for the ε4 allele. Specifically, larger waist circumference has been associated with lower fMRI signal in the superior frontal and middle frontal gyrus, and slower reaction times on a n-back working memory task among middle-aged/older adults (Gonzales et al., 2014). Similarly, middle-aged individuals with obesity show reduced brain activation in the right parietal cortex relative to individuals with normal-weight and overweight on a n-back working memory task (Gonzales et al., 2010). As mentioned above, these patterns are in contrast to those observed among ε4 carriers without overweight or obesity, with ε4 carriers tending to exhibit elevated brain activation during cognitive tasks compared to non-carriers (Bookheimer et al., 2000; Wishart et al., 2006; Chen et al., 2013; Scheller et al., 2017).

Considering the high prevalence of obesity, it is likely that many individuals with overweight/obesity carry at least one ε4 allele. Despite this, to our knowledge, no published study has examined brain activity of APOE ε4 allele carriers with overweight/obesity during a working memory task, let alone while controlling for potentially confounding factors such as body weight and cerebral perfusion. Accounting for CBF and measures of overweight/obesity in a single fMRI study on working memory related brain activity among ε4 carriers may be especially important for several reasons. One is that, CBF changes have been observed among individuals with overweight/obesity and ε4 carriers (Thambisetty et al., 2010; Wierenga et al., 2013; Dorrance et al., 2014, Brandon et al., 2018; Hays et al., 2019), and CBF is inherently related to fMRI brain activity through the BOLD response (Havlicek et al., 2017). Similarly, changes in working memory performance and brain activity have also been observed among both ε4 carriers (Reinvang et al., 2010; Gu et al., 2017), and in overweight/obesity (Smith et al., 2011; Coppin et al., 2014; Yang et al., 2020).

As such, using a n-back working memory task along with a measure of resting cerebral perfusion in a sample of mostly midlife adults with overweight/obesity, who were relatively sedentary, but otherwise relatively healthy, we tested the following: (1) whether there are differences between ε4 carriers and non-carriers on task-related brain activity during a working memory (n-back) task, (2) whether genetic-related variation in brain activity relates to performance on the n-back working memory task, (3) whether controlling for resting state CBF would account for associations between the ε4 allele and working memory (n-back) task-related brain activity. We predicted that ε4 carriers would show greater activity in regions that support working memory performance during the n-back task relative to non-carriers. Based on prior work, we predicted that greater fMRI responses in frontal-striatal regions among ε4 carriers would be associated with better performance on the n-back task. Finally, we hypothesized that controlling for CBF would not suppress any associations between the ε4 allele and working memory related brain activity.

## Materials and methods

### Participants

Participants included in the present study were drawn from a larger sample of individuals enrolled in a 12-month randomized clinical trial examining the impact of energy-restricted diet and exercise on cardiovascular health outcomes among adults with overweight or obesity (ClinicalTrials.gov NCT01500356; R01HL103646; PI: Jakicic) (Jakicic et al., 2022). Of the 383 individuals enrolled in the intervention, 125 (32.6%) consented to neuroimaging assessments. Individuals who participated in the neuroimaging arm of the study did not differ from the parent intervention sample on any clinical or demographic indicator, with the exception of systolic blood pressure; however, this difference was not clinically meaningful [parent sample, 120.2 (11.7) mm Hg; neuroimaging sample, 118.4 (11.7) mm Hg]. To be eligible for the parent trial, individuals had to be between the ages of 18 and 55 and have a BMI in the overweight or obese range from 25.0 to <40.0 kg/m^2^. As previously reported, participants were ineligible for the trial if they reported: (1) engagement in >60 min per week of structured moderate-to-vigorous intensity physical activity, (2) weight loss of >5% within the prior 6 months or a history of bariatric surgery, (3) history of cardiometabolic disease, diabetes mellitus, or cancer, (4) taking medication that could affect heart rate or blood pressure, (5) taking medication that could influence body weight, (6) treatment for psychological conditions that included medication or counseling, (7) currently pregnant, pregnant within the prior 6 months, or planning a pregnancy within the next 12 months, (8) planning on geographical relocation outside of the region within 12 months, or (9) inability to comply with the components of the interventions (Rogers et al., 2019; Jakicic et al., 2022). Participants also had to be free of any contraindication that would prohibit MRI scanning for both the parent and the brain ancillary project that we describe here. The present study focused on data collected during baseline assessments, which were completed within one month of the start of the intervention. Data for the neuroimaging arm of the study were collected between November 2013 and July 2016. All study procedures were approved by the University of Pittsburgh Institutional Review Board, and all participants provided informed consent before initiation of any procedures.

A subset of 100 individuals in the neuroimaging arm of the trial (80%) provided a saliva sample for genotyping (10 refused, 12 were unable to be contacted). From the group of participants with available genetic information, 24 possessed at least one APOE ε4 allele (21 ε3/ε4 carriers; 3 ε4/ε4 carriers). To minimize between group variability, these individuals were matched to a subsample of 24 non-carriers on factors that may influence the outcomes of interest such as age, years of education, and BMI. APOE ε4 allele carriers were matched to a non-carrier who was within ± 5 years for age, ± 2 years for years of education, and within ± 5 kg/m^2^ for BMI. The average difference between ε4 carriers and non-carriers matched pairs for age, years of education, and BMI was 0.58 years, 0.13 years, and 0.62 kg/m^2^, respectively. Race/ethnicity and sex were secondarily considered in matching; however, age, years of education, and BMI was used as the primary basis for matching ε4 carriers to non-carriers.

### Apolipoprotein E genotyping

Apolipoprotein E genotype was determined using the i-PLEX Gold single nucleotide polymorphism (SNP) assay, which relied on DNA that was extracted from saliva samples. For the purpose of our analyses, the APOE genotype was dichotomized, such that ε4 non-carriers were assigned a value of 0 and ε4 carriers received a value of 1.

### Working memory task

Working memory was assessed using a n-back task, which consisted of alternating blocks of 1-back and 2-back conditions interleaved with blocks of a visual crosshair acting as a baseline period (Peven et al., 2020). During this task, participants were asked to identify whether the current letter was the same as the letter that immediately preceded it (1-back) or two letters before (2-back). Participants pressed a button in the case of a match and a different button in the case of a non-match. In each block, a series of 16 letters were presented for 1.5 s (1-s inter-stimulus interval). This task consisted of six task blocks (three 1-back and three 2-back) for a total of 48 trials per condition that were interleaved with a visual crosshair period for the same length of time as the task blocks. Participants’ performance was evaluated in terms of accuracy in determining match and non-match trials.

### Neuroimaging protocol

#### Acquisition

As previously reported (Donofry et al., 2020; Aghjayan et al., 2021; Stillman et al., 2021), A Siemens 3.0 Tesla MR Verio system (Munich, Germany) with a 32-channel head coil was used for MR sequence data collection. High resolution, T1-weighted images were acquired with the following sequence parameters: Magnetization Prepared Rapid Acquisition of Gradient Echo (MPRAGE), matrix = 256, field-of-view (FOV) = 250 mm, voxel size = 1.0 × 1.0 × 1.0 mm, slices = 192 (sagittal plane, acquired left to right), slice thickness = 1.0 mm, repetition time (TR) = 1,900 ms, echo time (TE) = 2.93 ms, inversion time (TI) = 900 ms, flip angle = 9°, sequence duration = 4:26 min. Functional MRI images for the n-back task were acquired using echo-planar imaging with BOLD contrast (TR = 2,000 ms, TE = 30 ms, field of view = 24 × 24 cm, 64 × 64 matrix, 42 axial slices, 3.5 × 3.5 × 3.2 mm slice thickness, 0.3 mm gap, slices = 34). Perfusion-weighted images were collected using a multi-slice pseudo-continuous arterial spin labeling (pcASL) sequence with the following parameters (Jung et al., 2010): matrix size = 64, FOV = 220 mm, voxel size = 3.40 × 3.40 × 5.0 mm, slices = 20 (axial plane, acquired in ascending order), slice thickness = 5.0 mm, gap between slices = 1 mm, single slice acquisition time = 48 ms, label duration = 1,500 ms, post-label delay = 1,500 ms, TR/TE = 4,090/21 ms, volumes = 80, number of label/control pairs = 40, flip angle = 90°, RF blocks = 80, RF pulses = 20, gap between pulses = 360 μs, bandwidth = 2,298 Hz/Px, and sequence duration = 5:35 min.

### Neuroimaging analyses

#### Functional magnetic resonance imaging data preprocessing

Functional magnetic resonance imaging data were preprocessed with tools available from FMRIB’s Software Library 5.0.8 (FSL) fMRI Expert Analysis Tool 6.0 (FEAT) (Woolrich et al., 2001, 2009). The following pre-processing steps were conducted: motion correction using MCFLIRT (Jenkinson et al., 2002), removal of non-brain tissue using BET (Smith, 2002), spatial smoothing using a Gaussian kernel of full-width at half-maximum (FWHM) of 7.0 mm, and high-pass temporal filtering at a threshold of 150.0 s. Functional images were registered to participant’s T1-weighted MPRAGE and the Montreal Neurological Institute (MNI) 152 standard space template using 7- and 12-parameter affine transformations, respectively. Registration quality was visually inspected, and no registration errors were identified.

### Functional magnetic resonance imaging general linear model analysis

After completing the preprocessing and registration steps, we used FEAT in a first-level statistical analysis of the n-back task. Linear contrasts were created to examine activation in each of the task conditions, as follows: 1-back > rest, 2-back > rest, and 2-back > 1-back. Each contrast generated a statistical parametric map. These maps were used for higher-level mixed-effects whole-brain group analyses and ROI analyses (see below) using FMRIB’s Local Analysis of Mixed Effects (FLAME) (Beckmann et al., 2003).

### Whole-brain analysis

We first conducted a whole-brain omnibus analysis that tested for (1) brain regions where ε4 carriers had greater activation and (2) brain regions where ε4 carriers exhibited less activation than non-carriers during the n-back task (*n* = 48). Our group-level maps were thresholded using a voxel-wise *z*-score of 2.3 and a cluster threshold of *p* < 0.05.

#### ROI analysis

We conducted an ROI analysis to examine whether the regions identified in the whole-brain voxel-wise analysis for the contrast between ε4 carriers and non-carriers were related to either n-back performance or CBF. Specifically, we created 10 mm spheres around statistical peaks of activation from the voxel-wise whole-brain examination and then extracted the mean percent signal change within each of these spherical ROIs for each participant. These data were imported into SPSS for evaluating associations between fMRI activity, n-back performance and CBF.

### Pseudo-continuous arterial spin labeling data preprocessing and blood flow quantification

Following reconstruction, the first four volumes of the image time-series were discarded to assist in signal stabilization (Stillman et al., 2021). After removing these volumes, the middle volume was used as a reference and the data were co-registered with the participant’s T1-weighted anatomical image and to MNI space (FLIRT; FSL’s Linear Registration Tool, Oxford, UK) (Jenkinson and Smith, 2001; Jenkinson et al., 2002). Partial volume estimates were derived from the T1-weighted anatomical image using FSL’s Fully Automated Segmentation Toolbox (FAST) (Zhang et al., 2001). Finally, we used these high-resolution tissue-type maps for partial volume correction, nuisance signal regression, and tissue-specific CBF quantification (Stillman et al., 2021). Additional details on the preprocessing procedures for the pCASL data can be found in a previous report (Stillman et al., 2021).

To quantify CBF, we used tools available from FSL’s Bayesian Inference for Arterial Spin Labelling toolbox (BASIL) (Chappell et al., 2009). First, CBF images were obtained from pairwise tag-control subtraction of the co-registered CBF time-series. These images were adjusted for slice-time delay and BASIL’s oxford_asl command was run with the partial volume and spatial correction options turned on to help control for spurious signals (Groves et al., 2009; Chappell et al., 2011). We used the cerebral spinal fluid image from FAST as the reference tissue for nuisance regression. The CSF was used to compute the magnetization equilibrium (M0) of the white and gray matter tissue, which was then used to estimate the M0 of the arterial blood and convert the relative CBF values into absolute units of ml/100 g/min (asl_calib). These calibrated images were normalized to MNI space. The calibrated and normalized perfusion images from each person were combined into a 4D file for group-level analyses (Stillman et al., 2021). For our group-level analyses, we constructed a general linear model (glm_gui) to test for areas where (1) ε4 carrier CBF > non-carrier CBF and (2) non-carrier CBF > ε4 carrier CBF. The model was run using FSL’s flameo command, and results were corrected for multiple comparisons using FSL’s cluster tool at a threshold of *p* < 0.05.

### Planned analyses

Analysis sample consisted of 24 participants of the HEART HEALTH trial neuroimaging ancillary study who were ε4 carriers and another 24 non-carriers who were matched based on age, years of education, and BMI. To test for difference in working memory task-related brain activity between ε4 carriers and non-carriers, we conducted a whole-brain analysis of the n-back data by modeling a contrast between ε4 carriers and non-carriers for each condition of the n-back task. Second, after identifying ROIs that significantly differed between ε4 carriers and non-carriers, we conducted one-way ANOVA to address our second and third aims. To address our second aim and determine whether genetic-related variation in brain activity relates to performance on the n-back working memory task including the following four outcomes we conducted four sets of regression analyses stratified by APOE genotype. Each model differed with respect to the outcome variable, such that we evaluated whether fMRI brain activity for each ROI that statistically differed between ε4 carriers and non-carriers predicted: (1) accuracy during the 1-back condition, (2) reaction time during the 1-back condition, (3) accuracy during the 2-back condition, (4) reaction time during the 2-back condition. Regression models controlled for race/ethnicity, years of education, BMI, and sex. Finally, to address our final aim and determine whether any differences in CBF influenced fMRI activity between ε4 carriers and non-carriers, we conducted an ANCOVA controlling for CBF in each of the ROIs.

## Results

### Participant characteristics

Demographic characteristics for each group are presented in Table 1. In brief, ε4 carriers and non-carriers were intentionally matched on demographic variables (i.e., age, years of education, and BMI) and therefore did not significantly differ with regards to these measures. Additionally, demographic characteristics of the subsample presented in Table 1 did not significantly differ from the remainder of the neuroimaging sample (*p*s > 0.05).

**TABLE 1:** Demographic and n-back performance data for participants.

	APOE ε4+ (*n* = 24)	APOE ε4− (*n* = 24)	Remaining sample (*n* = 77)	APOE ε4+ vs. APOE ε4− *P*-value
Age	45.44 (8.90)	44.88 (8.14)	44.32 (8.79)	0.81
Female (%)	75%	83%	77%	0.49
Race/ethnicity *n* (% White)	16 (67%)	17 (70%)	58 (75%)	0.81
Years of education	16.21	16.08	16.49	0.87
BMI	32.43 (4.34)	32.22 (4.1)	30.91 (3.68)	0.61
1-Back accuracy	0.93 (0.10)	0.91 (0.16)	0.89 (0.24)	0.72
1-Back reaction time	879.32 (151.17)	890.24 (145.37)	871.10 (165.29)	0.80
2-Back accuracy	0.83 (0.13)	0.81 (0.18)	0.79 (0.26)	0.65
2-Back reaction time	1,009.95 (174.34)	1,015.53 (145.71)	1,060.17 (176.54)	0.90

APOE ε4+ indicates an ε4 carrier; APOE ε4− indicates a non-carrier; BMI, body mass index.

### Functional magnetic resonance imaging brain activation

**FIGURE 1:**
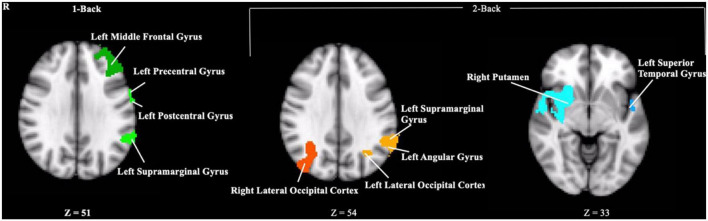
Clusters where ε4 carriers exhibited reduced activity during the n-back task compared to non-carriers. Each cluster met a cluster threshold of *z* > 2.3 and *p* < 0.05. These clusters are described further in Table 2.

**FIGURE 2:**
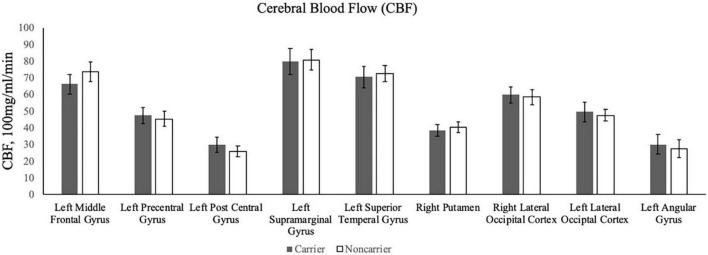
The bar graphs display mean CBF for the ε4 carriers and non-carriers in the ROIs derived from the whole-brain analysis of the n-back data.

Contrary to our hypothesis, ε4 carriers had significantly lower activation than non-carriers during the 1-back and 2-back conditions relative to the resting crosshair periods (refer to Figure 1 and Table 2) in numerous brain regions including areas of the prefrontal cortex, superior parietal cortex, lateral temporal lobe, occipital lobe, and basal ganglia. To account for any confounding effects of diminished CBF in ε4 carriers, we ran one-way ANCOVAs between genotype on n-back activation controlling for regional CBF in each ROI to compare between ε4 carriers and non-carriers. All of the results remained significant when controlling for CBF (see Figure 2 for mean CBF for carriers and non-carriers showing no group differences in CBF). Importantly, there were no significant group differences for the 2-back > 1-back contrast.

**TABLE 2:** Results of the whole-brain analysis of the n-back data modeling APOE genotype group differences in activation during the n-back.

Regions	Maximam *z*-score	Peak *X* (MM)	Peak *Y* (MM)	Peak *Z* (MM)	Signal change ε 4 carriers (%)	Signal change non-carriers (%)	*P*-value
Left middle frontal gyrus^1^	3.56	−47	32	26	0.26 ± 0.50	0.62 ± 0.31	<0.01
Left precentral gyrus^1^	2.86	−61	5	27	0.08 ± 0.53	0.41 ± 0.40	<0.05
Left postcentral gyrus^1^	2.52	−63	4	27	0.13 ± 0.75	0.63 ± 0.78	<0.05
Left supramarginal gyrus^1^	3.51	−61	−46	33	0.01 ± 0.35	0.24 ± 0.30	<0.05
Left superior temporal gyrus^1^	2.98	−57	−33	11	−0.17 ± 0.24	0.07 ± 0.25	<0.01
Right putamen/insula^2^	3.45	28.5	6	−9.5	0.08 ± 0.16	0.27 ± 0.19	<0.001
Right lateral occipital cortex^2^	3.82	31	−70	37	0.16 ± 0.33	0.59 ± 0.28	<0.001
Left lateral occipital cortex^2^	2.70	−31	−63	33	0.19 ± 0.25	0.42 ± 0.32	<0.05
Left angular gyrus^2^	3.07	−60	−51	37	−0.04 ± 0.51	0.42 ± 0.32	<0.001
Left supramarginal gyrus^2^	2.99	−61	−46	33	−0.07 ± 0.30	0.23 ± 0.36	<0.01

Regions were created from 10 mm spheres generated around the peak activation from the voxel-wise and cluster thresholded maps from the whole-brain analysis. This analysis did not include covariates.

^1^Regions significantly different during the 1-back condition (*p* < 0.05).

^2^Regions significantly different during the 2-back condition (*p* < 0.05).

### N-back working memory performance

Consistent with prior research of the APOE genotype in this age range, neither accuracy rates nor reaction times for either the 1-back or 2-back conditions were significantly different between ε4 carriers and non-carriers (refer to Table 1).

### Linking functional magnetic resonance imaging brain activation and n-back performance

To examine the association between fMRI brain activation during the n-back working memory task and performance, we extracted the fMRI signal from the ROIs described above. The benefit of this approach is that it focuses the analyses on the regions that were identified at the voxel-wise level that differed between ε4 carriers and non-carriers and thereby reduces the number of multiple comparisons. Among ε4 carriers, fMRI percent signal change for each ROI was not significantly related to reaction time during the 1-back or 2-back conditions (*p*s > 0.05). Similarly, there was no association between activation in any of the ROIs and accuracy rates for the 1-back condition or 2-back condition (*p*s > 0.05) among ε4 carriers. Similarly, among non-carriers, activation in the ROIs was not significantly correlated with either reaction time or accuracy during the 1-back or 2-back conditions (*p*s > 0.05).

### Cerebral blood flow

There were no significant differences in CBF for the comparison between ε4 carriers and non-carriers in each ROI. As described above, when including CBF as a covariate, the fMRI results between ε4 carriers and non-carriers did not change indicating that differences in CBF cannot explain the genetic differences in fMRI activity.

### Linking body mass index to functional magnetic resonance imaging brain activation and n-back performance

Testing whether BMI was associated with working memory related fMRI brain activity or working memory performance was not a primary aim of the current study. However, to probe the association between BMI and fMRI brain activation, we conducted a whole-brain analysis of the n-back data by examining brain regions in which BMI was associated with greater activation, and lower activation, for each condition of the n-back task. Using a voxel-wise *z*-score of 2.3 and a cluster threshold of *p* < 0.05, BMI was not associated with activation during any condition of the n-back task. Similarly, BMI was not associated with accuracy or reaction time during the 1-back or 2-back conditions (*p*s > 0.05).

### Body mass index and apolipoprotein E interaction on functional magnetic resonance imaging brain activation and n-back performance

We explored whether APOE genotype moderated any association between BMI and fMRI activity. To test this, we conducted a whole-brain analysis of the n-back data and examined brain regions in which BMI and APOE statistically interacted with level of fMRI activation. Using a voxel-wise *z*-score of 2.3 and a cluster threshold of *p* < 0.05, the BMI × APOE genotype interaction term was not associated with activation during any condition of the n-back task. Similarly, the APOE genotype and BMI interaction term was not associated with accuracy or reaction times during the 1-back or 2-back conditions of the n-back task (*p*s > 0.05).

## Discussion

The present study examined whether the APOE ε4 allele was associated with working memory-related neural activity in sedentary midlife adults with overweight or obesity. We predicted that (1) ε4 carriers would have greater fMRI responses during a n-back working memory paradigm, (2) greater activation would be associated with better task performance for ε4 carriers. Contrary to our hypothesis, we found that ε4 carriers had lower fMRI responses than non-carriers in several brain regions across frontal, parietal, temporal, occipital, and striatal regions during the 1-back and 2-back conditions, including the left middle frontal gyrus, left precentral gyrus, left postcentral gyrus, left supramarginal gyrus, right putamen, right lateral occipital cortex, left lateral occipital cortex, and left angular gyrus. In contrast to Fleisher et al. (2009) who found that higher baseline CBF accounted for lower fMRI brain activity among ε4 carriers, we did not observe any significant differences in CBF (see Figure 2). As such, we can conclude that the association between the ε4 allele and task-fMRI activation was statistically independent of differences in CBF in our sample of adults with overweight/obesity, but who were otherwise relatively healthy. Additionally, among ROIs that significantly differed between ε4 carriers and non-carriers, activation was not associated with reaction time nor accuracy during the n-back task.

We observed no significant differences between ε4 carriers and non-carriers in working memory task performance, a finding that is in line with several other studies of cognitively normal middle-aged ε4 carriers (Wishart et al., 2006; Chen et al., 2013) and cognitively normal older adult ε4 carriers (Scheller et al., 2017). This pattern has been interpreted as reflecting changes in brain function that are not yet manifest in behavioral outcomes such as reaction time measures and accuracy rates. Yet, the lack of an association with behavioral patterns makes the interpretation of the differences in brain activity more challenging because of the lack of a variable or outcome that could be used to anchor the results in a behavioral outcome.

The pattern of ε4 related fMRI activation observed in this study is different than other studies, which have reported increased task-related activation among cognitively normal ε4 carriers in this age range (Bookheimer et al., 2000; Wishart et al., 2006; Chen et al., 2013; Scheller et al., 2017). One possible explanation for this difference is that our sample is comprised entirely of individuals with overweight and obesity. Prior studies have not reported measure of overweight/obesity status in the context of the patterns of their results. Assuming prior studies were comprised of mostly normal weight individuals, or a combination of normal weight to overweight/obese individuals, our findings suggest that the presence of the ε4 allele may exert an effect on task-fMRI neural activity that differs as a function of obesity. However, this possible interpretation is weakened since we failed to find significant interactions between APOE genotype and BMI on brain activity patterns. Yet, low power might also be contributing to the lack of a statistically significant interaction term to adequately test this hypothesis. The interpretations and behavioral relevance of our findings are further questioned by the lack of associations between n-back fMRI activity and task performance. Such lack of an association between activity and performance is not unique to this study but it does raise questions about whether such activation differences are immaterial with respect to behavior or whether the activation differences reflect a pattern that might be predictive of longitudinal changes in performance.

Other studies have found that fMRI brain activity among ε4 carriers may confer increased risk for AD and the presence of AD-related pathology, particularly Aβ. Several fMRI studies report hypoactivation of regions that show early increases in pathology, including prefrontal and medial temporal regions among older adults with mild cognitive impairment (Johnson et al., 2006a; Petrella et al., 2006) and cognitively normal middle age/older adults with elevated levels of Aβ (Foster et al., 2018; for review see Sperling et al., 2010). The sample in the present study is relatively young and ε4 carriers did not display performance deficits on the n-back working memory task, which may indicate alterations in other underlying pathways related to brain health rather than frank cognitive impairments as observed among individuals with AD.

The mechanisms linking APOE to variation in brain function are not well understood, however; prior research suggests that altered cholinergic or dopaminergic functioning may be pathways through which APOE gene variation impacts fMRI related brain activity (Reverte et al., 2016). Indeed, alterations in dopaminergic function have been found to influence the hemodynamic response and neurovascular coupling, the process by which neural activity elicits cerebrovascular changes to fulfill metabolic demands of neurons (for review see Bruinsma et al., 2018). Dopamine is heavily implicated in the fronto-striatal network, which is comprised of many of the structures that ε4 carriers exhibited reductions in working memory-related brain activity. As such, dopaminergic system dysfunction may relate to the lower activity observed in prefrontal and the striatal regions, including the middle frontal gyrus, precentral gyrus, postcentral gyrus, and putamen.

Although dopaminergic alterations may be a plausible explanation for differences in activity in regions of the fronto-striatal network, reduced activity observed in the lateral occipital cortex and angular gyrus of ε4 carriers may be better explained by reductions in cerebral glucose metabolism. Not only has the ε4 allele been shown to play an important role in lipid metabolism more generally (Dupuy et al., 2001), but this also seems to extend to how the brain metabolizes glucose as reductions in cerebral glucose metabolism have been detected in ε4 carriers as early as their 20–30 s in the absence of cognitive deficits (Reiman et al., 2004). While glucose metabolism has been found to associate with Aβ (Furst and Lal, 2011; Carbonell et al., 2016), the ε4 allele may exert an effect on brain physiology independent of Aβ. This idea has been supported by a study that reported that the ε4 allele, but not Aβ, was associated with reduced cerebral glucose metabolism (Jagust et al., 2012).

### Strengths and limitations

The current study leveraged cross-sectional data from a large randomized clinical trial of mostly midlife adults with overweight/obesity. Additionally, data from this trial provided advanced multimodal neuroimaging data and genetic data which allowed us to test our aims. Unique to this study is that it examined differences in task-fMRI brain activity between midlife ε4 carriers and non-carriers matched on overweight/obesity, age, and years of education, as well as one of the first to control for a possible confounding factor (CBF), there are several limitations that must be considered.

First, while an effort was made to match the sample based on age, years of education, and BMI using a range of ± 5 years for age, ± 2 years for years of education, and ± 5 kg/m^2^ for BMI, future studies may benefit by using a smaller *a priori* cut off criteria for matching purposes. Similarly, sex and race/ethnicity did not significantly differ between ε4 carriers and non-carriers, but more deliberate matching on these characteristics could also eliminate potential confounding by these differences. In this study we were able to make a direct match on 83% of the cases by sex and 79% of the cases for race/ethnicity and the intent of the study was not to test for sex differences. While little work has been done to determine the extent to which sex and race/ethnicity differences may impact fMRI brain activity during the n-back task, the current study may have benefited by having more participants to allow for even greater matching on age, years of education, and BMI in addition to matching on sex and race/ethnic characteristics. Furthermore, conducting additional analyses to explore the independent effects that sex and race/ethnicity have on fMRI brain activity during the n-back working memory task may be useful to elucidate their importance as potential demographic features for matching. Future research should determine whether sex and race/ethnicity act as additional moderators by designing studies that would *a priori* match for these participant characteristics across ε4 carriers and non-carriers.

Another important consideration is that the lack of findings regarding n-back working memory performance may be attributable to the task conditions not being difficult enough. Indeed, accuracy rates were around 0.90 and 0.80 across the 1-back and 2-back conditions respectively. Future studies may benefit by incorporating conditions of the n-back task that require a greater working memory load, such as a 3-back condition, and/or evaluating a wider variety of working memory tasks to more holistically capture working memory function.

Another limitation is that we had a relatively small sample size. While evaluating brain activity among ε4 carriers with overweight/obesity was interesting and novel, a larger sample would have provided greater statistical power to determine the associations between BMI and working memory related brain activity among ε4 carriers, and we may have been more sufficiently powered to detect APOE genotype × BMI interactions on working memory related brain activity. Instead, we primarily characterized the impact of the ε4 allele on working memory related brain activity among individuals with overweight/obesity by matching individuals on BMI across APOE genotype group. A more nuanced focus on BMI could have been informative given that greater BMI has additive effects on other indicators of brain health, including cognitive functioning, brain volume, and white matter integrity (Zade et al., 2013; Rajan et al., 2014). Along these lines, we excluded individuals with normal-weight in this study, which prevented us from knowing how this sample is performing relative to adults with normal-weight.

We also lacked observational longitudinal data (i.e., data that demonstrates functioning of the sample into late adulthood) in this sample. Therefore, we were unable to determine the current or long-term impact of reduced task-fMRI brain activity on neurocognitive health. Indeed, while the reduced task-fMRI brain activity among ε4 carriers was interpreted as potentially maladaptive given the co-occurrence of metabolic and genetic risk, lower activation in the context of similar cognitive performance to non-carriers could represent neural efficiency for ε4 carriers such that they could perform similarly while using fewer neural resources. It is also important to consider that up to 63% of ε4 carriers may never develop AD (Crean et al., 2011), so the lack of observational longitudinal data makes it difficult to determine the extent to which the lower task-fMRI brain activity observed among ε4 carriers in the current study is related to risk of future development of AD.

Another limitation of the present study was the use of a cross-sectional design, which allows for the possibility that individual characteristics other than APOE genotype may be related to differences in brain activation. Although the ε4 allele has been consistently shown to be related to differences in brain activation, it is important to acknowledge that other individual factors have been shown to influence the association between the ε4 allele and neurocognitive function such as race/ethnicity (Barnes et al., 2013) and these may also be influencing the relationship between ε4 allele and fMRI brain activity. Furthermore, epigenetic factors could be influencing APOE gene expression, which could further complicate the impact of the ε4 on fMRI brain activity.

Finally, the present study lacked data on Aβ deposition and family history of AD, both of which have been associated with variation in fMRI brain activity in other studies (Johnson et al., 2006b; Foster et al., 2018; Kennedy et al., 2018). Such data may have (1) provided insight into what accounts for differences in fMRI brain activity among ε4 carriers with overweight/obesity and (2) further shown that alterations in fMRI brain activity among ε4 carriers with overweight/obesity may indicate additional risk for AD and neurocognitive decline in late adulthood.

## Conclusion

In conclusion, our findings demonstrate that in this mostly midlife age range of participants with overweight/obesity, we can detect significant differences in working memory-related brain activity as a function of APOE genotype. Our results suggest that these differences in brain activation are not related to a common comorbid condition in midlife that also elevates risk for neurocognitive decline – overweight/obesity – given that all participants were matched for BMI. We can also rule out the likelihood that differences in task-related working memory brain activity were confounded by differences in CBF. Finally, differences in brain activation did not translate to differences in performance on the n-back task. Although the APOE ε4 allele does not appear to be related to reduced performance on the n-back task, this does not preclude deficits in cognitive function on other tasks. Additionally, the findings of the current study do not rule out the possibility that ε4 carriers with overweight/obesity may be at increased risk for accelerated neurocognitive aging in late adulthood. However, longitudinal studies with larger sample sizes are required to determine the long-term impact of decreased neural activity, and to better characterize the individual and joint contributions of body fat and the ε4 allele in this rapidly growing and at-risk population. Such studies would allow researchers to pinpoint the pathophysiological basis for the brain activation patterns observed in ε4 carriers with overweight/obesity. Doing so could further promote the adoption of healthy lifestyle habits, such as physical activity, which has the potential to mitigate future neurocognitive declines (Erickson et al., 2011). Future research needs to clarify the long-term clinical implications of these findings with respect to brain health in the context of both overweight/obesity and genetic risk for AD. Nonetheless, our novel results could help inform policies to target individuals at greatest risk for dementia, well before the onset of clinical symptoms.

## Data availability statement

The data that support the findings of this study are available from JJ and KE, who were the Principal Investigators on the parent study and the ancillary study, respectively, upon reasonable request.

## Ethics statement

The studies involving human participants were reviewed and approved by the University of Pittsburgh Institutional Review Board. The patients/participants provided their written informed consent to participate in this study.

## Author contributions

JD, KE, and SA contributed to conception and study design. JJ, RR, WL, KE, KR, and SD contributed to data collection and acquisition. JD, SA, CS, SD, and KE contributed to statistical analysis and interpretation of results. JD and KE drafted the manuscript. JD, KE, JJ, SA, CS, WL, RR, SD, and KR critically revised the manuscript for important intellectual content. All authors approved the final version to be published and agreement to be accountable for the integrity and accuracy of all aspects of the work.
